# Significant variation in the assessment and management of screen-detected colorectal polyp cancers

**DOI:** 10.1007/s00384-024-04780-y

**Published:** 2024-12-22

**Authors:** Sally Hallam, Alexia Farrugia, David N. Naumann, Nigel Trudgill, Shantanu Rout, Sharad Karandikar

**Affiliations:** 1 University Hospitals Birmingham, Bordesley Green East, Birmingham, B9 5SS UK; 2 Sandwell and West Birmingham NHS Trust, Hallam Street, West Bromwich, B71 4HJ UK; 3 Institute of Cancer and Genomic Sciences, University of Birmingham, Birmingham, UK

**Keywords:** Colorectal polyp cancer

## Abstract

**Purpose:**

Endoscopic resection is appropriate for selected colorectal polyp cancers, but significant variation exists in treatment. This study aims to investigate variation in management of screen-detected polyp cancers (T1), factors predicting primary endoscopic polypectomy and threshold for subsequent surgical resection.

**Method:**

Patients with polyp cancers (T1) diagnosed by the bowel cancer screening programme (BCSP) were investigated at two screening centres (5 individual sites and 4 MDTs, 2012–2022). Patient demographics, pathological characteristics, management and outcomes were recorded. Variation in management was compared between sites. Risk factors for primary endoscopic polypectomy and the need for subsequent surgical resection were analysed using multivariable binary logistic regression models.

**Results:**

Of 220 polyp cancers, 178 (81%) underwent primary endoscopic resection. Secondary surgical excision was required in 54 (30%). Study sites were not significantly different in their primary management for colonic or rectal polyps. Only the size of colonic polyps was associated with primary surgery rather than endoscopic polypectomy (OR 1.05 (95% CI 1.00–1.11); *p* = 0.038). There was a difference between study sites in the odds ratio for secondary surgery after primary polypectomy for colonic polyps (OR 3.97 (95% CI 1.20–16.0); *p* = 0.033) but not rectal. Other factors associated with the requirement for secondary surgery were as follows: sessile morphology for colonic polyps (OR 2.92 (95% CI 1.25–6.97); *p* = 0.013) and en-bloc resection for rectal polyps (OR 0.14 (0.02–0.85); *p* = 0.043).

**Conclusion:**

There was significant variation in the assessment and treatment of colonic polyp cancers. Standardising pathology reporting and treatment algorithms may lead to better consistency of care and a reduction in secondary surgery.

## Introduction

The implementation of the UK’s national Bowel Cancer Screening Programme (BCSP) has led to an increase in the proportion of early-stage colorectal cancers (CRC) and a decrease in cancer-specific mortality [1]. Malignant colorectal polyps (T1 CRC; adenocarcinoma within the muscularis mucosa that may extend into but not beyond the submucosa) account for 10% of screen-detected CRC [1]. These can be completely excised endoscopically, or with conventional surgery. There is variation in the treatment of T1 CRC with wide variation in the use of primary surgery ranging from 7 to 36% [2].

The key to successful resection is an accurate endoscopic assessment. Increasing polyp size is correlated with the risk of malignancy, with polyps greater than 2 cm having a 40% chance of malignancy [4]. A number of classification systems predict the risk of submucosal invasion and successful endoscopic resection. These include the Kudo pit pattern, the Narrow Band Imaging International Colorectal Endoscopic Classification (NICE) and the Japanese Narrow Band Imaging Expert Team Classification (JNET) [5]. Polyp morphology is assessed by the Paris classification which describes pedunculated, sessile and flat lesions [5]. High-risk histological features may necessitate further treatment. These include the following: tumour differentiation, lymphovascular invasion, the presence of mucinous tumour, tumour budding [7] and the resection margin and the depth of submucosal invasion, as defined by the Haggitt or Kikuchi classification systems.

There is currently a lack of consensus in the endoscopic assessment of high-risk polyps, variation in histological reporting and the decision to proceed with endoscopic or primary surgical resection and the need to proceed to secondary surgical resection [5, 9, 10]. Managing oncological safety, patient preference and operative risk to achieve a safe resection without compromising quality of life or survival can be challenging.

The aim of the current study was to investigate the assessment and treatment of screen-detected T1 CRC over a decade (2012–2022) in a UK city that includes 4 hospital sites, including the decision-making regarding endoscopic or surgical treatment. We hypothesised that there would be significant variations in practice between sites even in the same city, and that these variations would be associated with differences in endoscopic assessment and pathology reporting and threshold to operate.

## Methods

### Study design and setting

A retrospective observational study was undertaken using prospectively collated data in the Open Exeter database of the BCSP. The database for the study included all consecutive BCSP patients in Birmingham, UK, from two screening hubs (at four hospital sites) over an 11-year period (January 2012 to December 2022). Institutional approval was obtained prior to data collection (Ref: 2594).

### Data collection

A data collection tool was used across each site to extract patient demographics (age, sex, Index of Multiple Deprivation (IMD; a scale of 1–10 where 1 was most deprived and 10 least deprived), frailty score (extrapolated from documented morbidity and frailty), endoscopic polyp assessment (size in mm, location, difficult access, Paris morphology, kudo pit pattern, Narrow Band Imaging, International Colorectal Endoscopic (NICE) polyp classification, Site, Morphology, Size, Access (SMSA) polyp level (extrapolated from endoscopy report), the presence of synchronous large polyp > 20 mm or synchronous cancer), the primary intervention (endoscopic polypectomy or primary surgery as the next intervention following the initial colonoscopy), radiological nodal staging, site, histological characteristics (Haggitt or Kikuchi level, resection margin in mm, differentiation, mucinous, tumour budding, lymphovascular invasion, histological TNM), secondary surgical intervention (segmental colectomy or local excision of incompletely resected or polyps at high risk of recurrence), for patients who had surgery Clavien-Dindo post-operative morbidity and mortality, as well as oncological outcomes of local and distant recurrence and overall survival.

### Outcomes

The outcomes of interest were primary endoscopic polypectomy and the requirement for subsequent surgery. These were considered to be important surrogate markers of variations in practice between sites.

### Data analysis

Demographics and polyp characteristics were compared across each MDT site using Kruskal–Wallis for continuous variables and chi squared for categorical variables. Outcomes according to site were compared using chi squared. Factors predicting primary endoscopic polypectomy and the need for secondary surgical resection were analysed using binary logistic regression models for the odds ratio and 95% confidence intervals for secondary surgical resection for patients who had primary polypectomy of (a) colonic and (b) rectal polyp cancers according to site, patient and polyp variables**.** For binary logistic regression, categorical variables were simplified to binary variables where possible, e.g. Kudo 4/5, and Paris classification simplified to pedunculated or sessile. IMD score and SMSA score were treated as continuous variables. First, univariate models were used with independent variables chosen as those likely to influence the outcomes. These included demographics (patient age, sex, deprivation, frailty score) and polyp characteristics (size in mm, location, Kudo pitt pattern, pedunculated or sessile, the presence of a synchronous polyp of greater than 20 mm or a synchronous cancer and radiological nodal status). Then the factors with a *p*-value of less than 0.05 were selected for the multivariable models. A *p*-value of < 0.05 was considered statistically significant.

## Results

### Study patient characteristics

There were 220 patients treated for screen-detected T1 CRC during the study period. Median age was 73 years (IQR 55–92), and 165 (75%) were male. Across the four sites, 46 (21%) were treated at site A, 49 (22%) at site B, 73 (33%) at site C (this site is the local centre for early rectal cancer surgery) and 52 (24%) at site D. Table 1 describes the patient demographics and variation according to site. Deprivation varied significantly by site with a higher proportion of the most deprived patients at sites C and D. Paris morphology was reported in 95% of patients, but site D had significantly reduced reporting of Paris morphology reported leading to a significant difference between sites. The surface pattern (Kudo) was reported for only 18% of patients.

**Table 1:** Demographics and polyp characteristics by site

Variable	All (*N* = 220)	A (*n* = 45)	B (*n* = 49)	C (*n* = 73)	D (*n* = 53)
**Age median (range)**	73 years (55–92)	73 (63–86)	74 (55–86)	71 (62–92)	73 (58–86)
**Male sex**, *n* (%)	165 (75)	40 (89)	37 (76)	51 (49)	37 (70)
**Deprivation, *n* (%)** 1 most 2 3 4 5 6 7 8 9 10 least	35 (16) 40 (18) 17 (8) 20 (9) 22 (10) 19 (9) 19 (9) 11 (5) 17 (8) 20 (9)	6 (13) 7 (16) 3 (7) 2 (4) 3 (7) 5 (11) 1(2) 2 (2) 8 (18) 8 (18)	8 (16) 18 (37) 10 (20) 4 (8) 5 (10) 4 (8) 0 (0) 0 (0) 0 (0) 0 (0)	12 (16) 5 (7) 1 (1) 6 (8) 8 (11) 5 (7) 12 (16) 7 (10) 9 (12) 8 (11)	9 (17) 10 (19) 3 (6) 8 (15) 6 (11) 5 (9) 6 (11) 2 (4) 0 (0) 4 (8)
**Frailty score, *n* (%)** 1 least 2 3 4 5 6 most	77 (35) 94 (43) 30 (14) 9 (4) 9 (4) 1 (0)	15 (33) 18 (40) 7 (16) 2 (4) 3 (7) 0 (0)	23 (47) 33 (67) 8 (16) 4 (8) 4 (8) 1 (2)	34 (47) 11 (15) 4 (5) 0 (0) 0 (0) 0 (0)	5 (9) 32 (60) 11 (21) 3 (6) 2 (4) 0 (0)
**SMSA, *n* (%)** 1 2 3 4	22 (10) 116 (53) 48 (22) 34 (15)	5 (11) 23 (51) 12 (27) 5 (11)	5 (10) 34 (69) 2 (4) 8 (16)	9 (12) 29 (40) 22 (30) 13 (18)	3 (6) 30 (57) 12 (23) 8 (15)
**Paris, *n* (%)** 1P 1S 1SP 2 2A 2B LST NS	71 (32) 95 (43 25 (11) 2 (1) 11 (5) 2 (1) 2 (1) 12 (5)	19 (42) 16 (36) 3 (7) 0 (0) 4 (9) 2 (4) 1 (2) 0 (0)	26 (53) 25 (51) 11 (22) 2 (4) 1 (2) 0 (0) 0 (0) 8 (16)	15 (21) 18 (25) 10 (14) 0 (0) 5 (7) 0 (0) 0 (0) 1 (1)	11 (21) 36 (68) 1 (2) 0 (0) 1 (2) 0 (0) 1 (2) 3 (6)
Size mm, median (range)	16 (1–65)	15 (1–65)	17.5 (4–10)	15 (5–60)	19 (4–55)
**Location**, *n* (%) Right Hepatic Transverse Splenic Descending Sigmoid Rectum	13 (6) 3 (1) 5 (2) 5 (2) 8 (4) 109 (50) 77 (35)	3 (7) 1 (2) 1 (2) 0 (0) 1 (2) 14 (31) 25 (56)	0 (0) 1 (2) 1 (2) 1 (2) 3 (6) 44 (38) 23 (18)	4 (5) 0 (0) 0 (0) 3 (4) 1 (1) 28 (38) 13 (18)	6 (11) 1 (2) 3 (6) 1 (2) 3 (6) 23 (43) 16 (30)
**Kudo 4 or 5, *n* (%)** Not specified 3 s 3 l 4 5	177 (80) 1 (0) 6 (3) 28 (13) 8 (4)	43 (96) 0 (0) 1 (2) 1 (2) 0 (0)	49 (100) 0 (0) 0 (0) 0 (0) 0 (0)	63 (86) 1 (1) 2 (3) 2 (3) 5 (7)	22 (42) 0 (0) 3 (6) 25 (47) 3 (6)
**Synchronous polyp > 20 mm**, *n* (%) No Yes	212 (96) 7 (3)	43 (96) 2 (4)	45 (92) 4 (8)	72 (99) 1 (1)	52 (98) 1 (2)
**Synchronous cancer**	2 (1)	0 (0)	0 (0)	0 (0)	2 (4)
**Nodal status** N0 N1	213 (97) 7 (3)	44 (98) 1 (2)	47 (96) 2 (4)	72 (99) 1 (1)	50 (94) 3 (6)

SMSA site, morphology, size, access

### Polyp characteristics

#### Endoscopic assessment

There was variation in the endoscopic polyp assessment parameters which were reported. Paris morphology was documented for 202 (95%), Kudo surface pit pattern for 40 (18%). NICE or JNET polyp classifications were not reported for any patient. The resection technique and whether the polyp was removed en bloc (e.g. EMR / ESD / hot or cold snare) were specified in all of the polyps endoscopically removed (178).

### Histological assessment

There was some variation in histological parameters reported. Haggitt or Kikuchi levels were reported for 118 (66%) of patients. For 22 (12%), the polyp was fragmented or did not include muscularis mucosa meaning it was not possible to report the Haggitt or Kikuchi level. For the remaining 16 (9%), the level was not reported. Differentiation was reported for 178 (all patients), lymphovascular invasion for 173 (97%), the presence of tumour budding for 32 (18%) and the presence of mucinous tumour for 25 (14%).

### Primary intervention

**Fig. 1:**
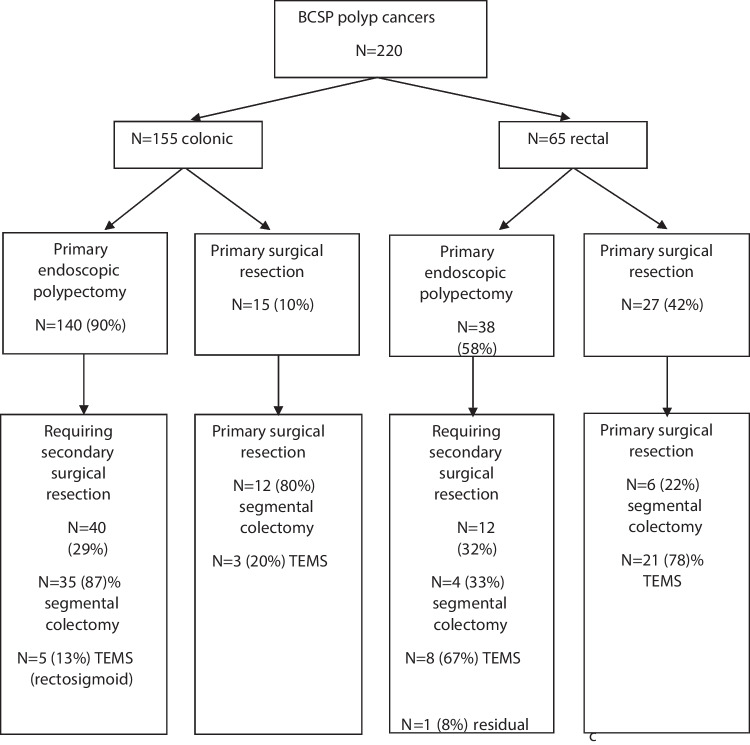
Primary and secondary polyp cancer management. BCSP Bowel Cancer Screening Program, TEMS transanal endoscopic microsurgery

Primary and secondary interventions are summarised in Fig. 1. Of the 220 patients, 155 had colonic and 65 rectal T1 CRC. Primary endoscopic polypectomy was performed for 178 patients (81%), which included 140 colonic (90%) and 38 (58%) rectal, all were performed with diathermy snare. The majority of endoscopic resections were en bloc in 144 patients (79%). Medilogik reporting software was used for endoscopy reporting across all four sites. The remaining 42 patients (19%) had primary surgery. Of those undergoing primary surgery, 25 (60%) had transanal endoscopic microsurgery (TEMS), and the remaining 17 (40%) had a segmental colectomy (Fig. 1). Following primary endoscopic resection, there were 2 local recurrences which were salvaged with a TEMS and a segmental colectomy, respectively. Patients with local recurrence all had an initial R0 endoscopic resection. There were 2 distant recurrences of which one died (Fig. 1).

### Surgical resection

Of those patients having a primary endoscopic intervention, 54 (30%) required a secondary surgical resection; this decision was determined by MDT and patient discussion. Segmental colectomy was performed for 38 (72%) and TEMS for 16 (28%). Of the patients having secondary surgical resection, 48 (89%) had no residual cancer on histological assessment of the specimen. Of the 6 patients with residual cancer in the resection specimen, 1 (2%) had lymph node involvement. The median length of stay for segmental colectomy was 4.5 days (range 1–12). There were no post-operative deaths. Morbidity with a Clavien-Dindo complication of 3 or above occurred in 2 patients; one anastomotic leak required interventional radiology (IR) drainage and one required return to theatre.

### Variation in polyp management by site

**Fig. 2:**
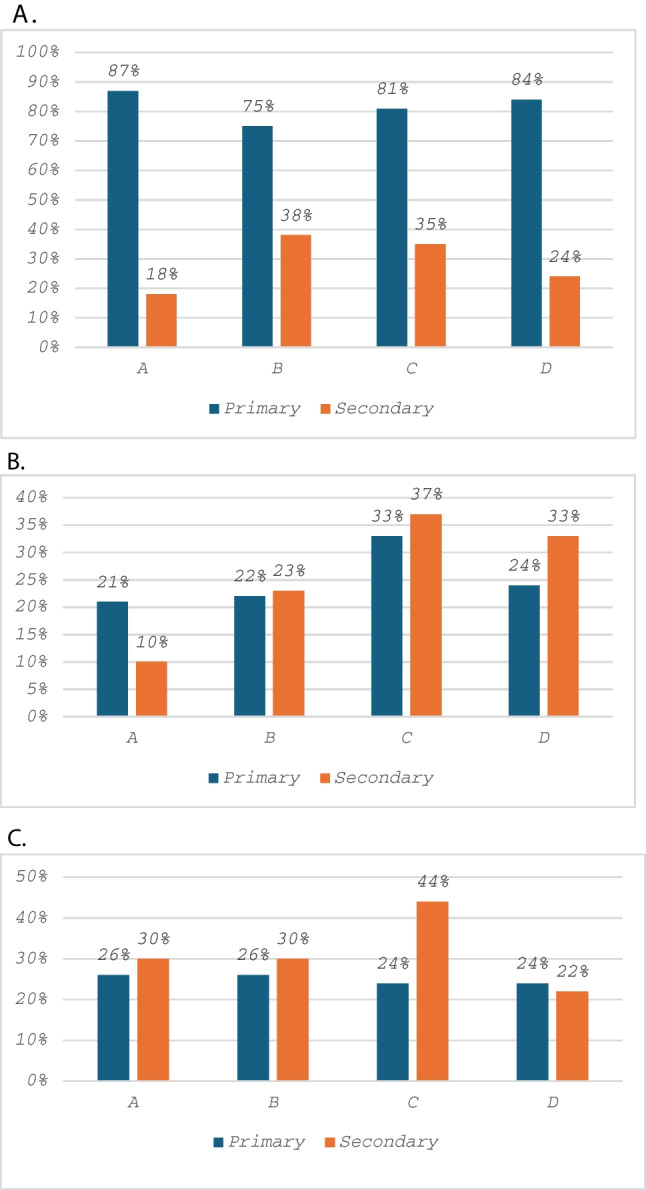
Variation in primary (endoscopic or surgical) polyp management and secondary surgical management for (**a**) all study patients; (**b**) colonic polyps and (**c**) rectal polyps according to site

There was no statistically significant variation in primary polyp management (endoscopic or surgical) by site, with 39 (87%) managed endoscopically at site A, 41 (85%) at site B, 55 (75%) at site C and 43 (81%) at site D, *p* = 0.445 (Fig. 2).

There was no significant variation in secondary surgical polyp management following primary endoscopic management by site with 7 (18%) at site A, 10 (24%) at site B, 21 (38%) at site C and 15 (35%) at site D, *p* = 0.134 (Fig. 2).

When patients were analysed according to anatomy (colonic or rectal T1 CRC), study sites were not significantly different in their primary management of T1 CRC for colon or rectal polyps (Table 2, Fig. 2). There was significant variation in secondary surgical management by site for colonic polyp cancers (3 (10%) at site A, 7 (23%) at site B, 17 (37%) at site C and 13 (33%) at site D; *p* = 0.038) (Table 3, Fig. 2). Study sites were not significantly different in their secondary surgical management of T1 rectal polyps (Table 2).

**Table 2:** Univariable and multivariable logistic regression models for the odds ratio and 95% confidence intervals for primary surgical resection of colonic (part A) andrectal (part B) polyp cancers according to site, patient and polyp variables

Variable of interest	(a) Univariable			(b) Multivariable		
	OR	95% CI	*p*-value	OR	95% CI	*p*-value
**A. Colonic polyp cancer**						
Site *(A* = *Ref)*						
* Site B*	2.34	0.46, 17.2	0.332	1.91	0.33, 15.5	0.487
* Site C*	1.26	0.23, 9.51	0.796	1.25	0.22, 9.99	0.806
* Site D*	1.71	0.31, 12.9	0.554	1.31	0.20, 11.1	0.732
Age	1.07	0.98, 1.17	0.144			
Male sex	2.25	0.58, 14.8	0.301			
IMD ≤ 3	0.92	0.29, 2.67	0.554			
SMSA ≤ 3	1.73	0.58, 5.11	0.316			
Sessile morphology	1.56	0.46, 5.21	0.464			
Size, mm	1.05	1.00, 1.11	0.040*	1.05	1.00, 1.11	0.038*
Kudo 4/5	0.78	0.08, 17.4	0.844			
Nodes positive	3.26	0.16, 27.5	0.320			
**B. Rectal polyp cancer**						
* Site B*	0.75	0.12, 4.28	0.745	0.71	0.11, 4.12	0.704
* Site C*	3.89	0.98, 17.9	0.063	3.04	0.72, 14.5	0.140
* Site D*	1.67	0.36, 8.42	0.519	1.23	0.24, 6.57	0.801
Age	1.06	0.97, 1.17	0.232			
Male sex	0.38	0.12, 1.18	0.098			
IMD ≤ 3	0.73	0.26, 1.98	0.535			
SMSA ≤ 3	1.85	0.68, 5.11	0.231			
Sessile morphology	0.81	0.27, 2.45	0.707			
Size, mm	1.05	1.00, 1.10	0.049*	1.04	0.99, 1.09	0.137
Synchronous large polyps	1.42	0.05, 37.1	0.806			
Nodes positive	2.96	0.27, 65.7	0.386			

*OR* odds ratio, *95% CI* 95% confidence interval, *IMD* index of multiple deprivation, *SMSA* size/morphology/site/access

**Table 3:** Univariable and multivariable logistic regression models for the odds ratio and 95% confidence intervals for secondary surgical resection for patients who had primary polypectomy of colonic (part A) andrectal (part B) polyp cancers according to site, patient and polyp variables

Variable of interest	(a) Univariable			(b) Multivariable		
	OR	95% CI	p-value	OR	95% CI	***p***-value
**A. Colonic polyp cancer**						
Site *(A* = *Ref)*						
* Site B*	1.82	0.49, 7.71	0.383	1.78	0.45, 7.84	0.419
* Site C*	3.67	1.17, 14.0	0.036*	3.97	1.20, 16.0	0.033*
* Site D*	3.87	1.17, 15.4	0.036*	2.69	0.76, 11.2	0.141
Age	0.98	0.93, 1.04	0.541			
Male sex	0.40	0.18, 0.88	0.023*	0.51	0.22, 1.21	0.124
IMD ≤ 3	0.96	0.45, 2.00	0.917			
SMSA ≤ 3	1.40	0.65, 2.99	0.380			
Sessile morphology	3.15	1.49, 6.81	0.003*	2.92	1.26, 6.97	0.013*
Size, mm	1.01	0.97, 1.06	0.598			
Kudo 4/5	0.44	0.07, 2.87	0.376			
Synchronous large polyps	1.22	0.16, 6.51	0.824			
En-bloc resection	0.73	0.30, 1.89	0.509			
Poor differentiation	3.56	0.75, 18.8	0.108			
Mucinous	0.75	0.03, 8.30	0.825			
Tumour budding	1.83	0.06, 52.3	0.687			
LVI	0.42	0.06, 1.68	0.280			
Resection margin < 1 mm	1.42	0.66, 3.00	0.362			
**B. Rectal polyp cancer**						
Site *(A* = *Ref)*						
* Site B*	1.00	0.14, 7.16	1.000	1.00	0.13, 7.85	1.000
* Site C*	1.87	0.28, 13.5	0.517	1.22	0.14, 10.1	0.850
* Site D*	0.67	0.07, 5.29	0.702	0.35	0.02, 3.41	0.385
Age	1.15	1.00, 1.35	0.060			
IMD ≤ 3	0.50	0.11, 2.01	0.341			
SMSA ≤ 3	3.80	0.92, 17.1	0.069			
Size, mm	1.06	0.99, 1.14	0.130			
En-bloc resection	0.18	0.03, 0.93	0.045*	0.14	0.02, 0.85	0.043*
Poor differentiation	0.77	0.04, 6.86	0.827			
LVI	2.75	0.43, 17.8	0.269			
Resection margin < 1 mm	0.45	0.09, 1.94	0.310			

*OR* odds ratio, *95% CI* 95% confidence interval, *IMD* index of multiple deprivation, *SMSA* size/morphology/site/access, *LVI* lymphovascular invasion

### Factors associated with primary endoscopic resection and secondary surgical resection

Study sites were not significantly different in their primary management of T1 CRC for colon or rectal polyps. Only the size of colonic polyps was associated with primary surgery rather than endoscopic polypectomy (larger polyps were associated with higher OR for primary surgery) (OR 1.05 (95% CI 1.00–1.11); *p* = 0.038). There was a difference between study sites in the odds ratio for secondary surgery after primary polypectomy for colonic polyps but not rectal. The only other factors associated with the requirement for secondary surgery after primary polypectomy were as follows: sessile morphology for colonic polyps (associated with increased likelihood of secondary surgery) (OR 2.92 (95% CI 1.25–6.97); *p* = 0.013) and en-bloc resection for rectal polyps (patients with en-bloc resection were less likely to require secondary surgery) (OR 0.14 (0.02–0.85); *p* = 0.043).

## Discussion

Primary endoscopic polypectomy only was performed for the majority of patients with good oncological outcomes. Study sites were not significantly different in their primary management of T1 CRC for colon or rectal polyps. There was a difference between study sites in the odds ratio for secondary surgery after primary polypectomy for colonic polyps but not rectal. The endoscopic and histological assessment of polyp cancers was variable. Of the known high-risk features, only the size of colonic polyps was associated with primary surgery rather than endoscopic polypectomy. Sessile morphology for colonic polyps was the only associated factor with secondary surgery, En-bloc resection was the only associated factor for rectal polyps.

A large proportion of patients in this cohort (81%) had primary endoscopic management of their colorectal polyp cancer, with low rates of local recurrence (1%). The literature suggests between 25.5 and 61% of colorectal polyp cancers are managed with primary endoscopic polypectomy [9, 12, 13], equivalent oncological outcomes for selected colorectal polyp cancers managed endoscopically [9, 12–14] and significantly lower morbidity 2.4% vs. 10.9% *p*-value < 0.001 [14].

There was significant variation in endoscopic polyp assessment and the classification systems used; the majority reported the Paris morphology 95%, and the Kudo surface pattern was documented in 18%, but no report documented the NICE or JNET classification. Variation in histological reporting was also noted with Kikuchi of Haggitt levels which were reported for 118 (66% of patients), differentiation for 100%, lymphovascular invasion for 97%, tumour budding for 18% and the presence of mucinous tumour for 14%. We hypothesise that differences in polyp assessment and reporting may influence primary polypectomy and secondary surgery, but this would require a larger cohort to test the hypothesis. Numerous potential classification systems are in use for endoscopic polyp assessment [5]. The sensitivity and specificity for these classification systems in predicting the depth of submucosal invasion vary, and their use varies by endoscopist, surgeon and centre preference. Shaukat et al. in the US Multi-society Task Force propose a guideline where sessile or large NICE III or Kudo V lesions are biopsied, tattooed and proceed directly to segmental resection and the remainder proceed to endoscopic resection [5]. Richards et al. made similar recommendations, and they found that only patients with incomplete resection at polypectomy or lymphovascular invasion were at risk of recurrence or cancer-related death [10].

The primary management of T1 CRC for colon or rectal polyps was not significantly different by study site secondary surgery after primary polypectomy differed by study site for colonic polyps but not rectal (Table 3). Only the size of colonic polyps was associated with primary surgery rather than endoscopic polypectomy (larger were associated with higher OR for primary surgery). There is a lack of standardised guidelines regarding the endoscopic management of colorectal polyp cancers and in particular the decision to proceed to secondary surgical resection based on the pathological findings. ACPGBI in 2018 developed a treatment algorithm for management using a cohort of BCSP polyp cancers, and they found the factors increasing the risk of residual tumour and lymph node metastasis and necessitating further surgery were incomplete resection and lymphovascular invasion [15]. Japanese guidelines from 2021 describe the endoscopic management of colorectal polyps including polyp cancers, and whilst they describe high-risk lesions, the guidelines do not describe when to proceed to subsequent surgical resection [16]. In a number of proposed guidelines, Symer et al. discuss the balance between oncological safety, patient preference and surgical and anaesthetic risk. They propose that if a polyp is removable endoscopically, it should then proceed to pathological assessment, high-risk lesions (SM3, Haggitt 4, poor differentiation, LVI, margin < 1 mm or not assessable), and sessile lesions of the distal rectum should proceed to secondary surgical resection [17]. Shaukat et al. recommend that non pedunculated polyps with deep submucosal invasion (NICE 3, Kudo V) should proceed directly to surgical resection, whilst pedunculated polyps should undergo endoscopic polypectomy [5]. High-risk histological features on pathological assessment (depth of submucosal invasion > 1 mm, positive polypectomy margins, poor differentiation, tumour budding and LVI) necessitate secondary surgical resection [5].

In this study, we found the only factors associated with the requirement for secondary surgery after primary polypectomy were as follows: sessile morphology for colonic polyps (associated with increased likelihood of secondary surgery) and en-bloc resection for rectal polyps (patients with en-bloc resection were less likely to require secondary surgery). There is clearly scope for guidelines detailing which high-risk features necessitate further surgical resection.

This study benefits from a large consecutive patient cohort across two bowel cancer screening centres in a single city. Whilst the variation in decision-making is captured for only four providers, it is likely that this is mirrored nationally.

## Limitations

Limitations of this study include the small cohort and its retrospective design, which means the capture of decision to proceed with endoscopic or secondary surgery relies heavily on the quality and detail of documentation in the endoscopy report and MDT outcome. It was unclear why some decisions were made and why some polyp classification systems were not used or why some were favoured over others. The multivariate analysis is complex due to the number of variables and is at risk of over-fitting.

## Conclusions

In this cohort, the majority of patients were treated with primary endoscopic management of their colorectal polyp cancer with favourable oncological outcomes. There was significant variation by MDT in the decision to proceed with secondary surgical resection. There is a need for guidelines to standardise management and optimise oncological outcomes whilst minimising patient surgical risk.

## Data Availability

Data is provided within the manuscript.

## References

[1] Logan RFA, Patnick J, Nickerson C, Coleman L, Rutter MD, von Wagner C (2012) Outcomes of the bowel cancer screening programme (BCSP) in England after the first 1 million tests. Gut 61:1439–1446 22156981 10.1136/gutjnl-2011-300843 PMC3437782

[2] Lee TJ, Rees CJ, Nickerson C et al (2013) Management of complex colonic polyps in the English Bowel Cancer Screening Programme. Br J Surg 100:1633–1639 24264787 10.1002/bjs.9282

[3] Kyzer S, Begin LR, Gordon PH, et al (1992) The care of patients with colorectal polyps that contain invasive adenocarcinoma. Endoscopic polypectomy or colectomy? Cancer 70:2044–2050. 10.1002/1097-0142(19921015)70:8<2044::aid-cncr2820700805>3.0.co;2-x 1394034

[4] Shinya H, Wolff WI (1979) Morphology, anatomic distribution and cancer potential of colonic polyps. Ann Surg 190:679–683 518167 10.1097/00000658-197912000-00001 PMC1345622

[5] Shaukat A, Kaltenbach T, Dominitz JA, Robertson DJ, Anderson JC, Cruise M, Burke CA, Gupta S, Lieberman D, Syngal S, Rex DK (2020N) Endoscopic recognition and management strategies for malignant colorectal polyps: recommendations of the US Multi-Society Task Force on Colorectal Cancer. Am J Gastroenterol 115(11):1751–1767 33156093 10.14309/ajg.0000000000001013

[6] Parker J, Gupta S, Shenbagaraj L, Harborne P, Ramaraj R, Karandikar S, Mottershead M, Barbour J, Mohammed N, Lockett M, Lyons A, Vega R, Torkington J, Dolwani S (2023F 3) Outcomes of complex colorectal polyps managed by multi-disciplinary team strategies-a multi-centre observational study. Int J Colorectal Dis 38(1):28 36735059 10.1007/s00384-022-04299-0 PMC9898359

[7] Brown I, Zammit AP, Bettington M, Cooper C, Gill AJ, Agoston A, Odze R (2023O) Pathological features associated with metastasis in patients with early invasive (pT1) colorectal carcinoma in colorectal polyps. Histopathology 83(4):591–606. 10.1111/his.14970 37366086 10.1111/his.14970

[8] Dolwani S (2019M) Significant polyp and early colorectal cancer - decision-making and treatment planning in regional networks and multidisciplinary teams. Colorectal Dis 21:16–18. 10.1111/codi.14492 30809917 10.1111/codi.14492

[9] Cooper GS, Xu F, Barnholtz Sloan JS, Koroukian SM, Schluchter MD (2012F 1) Management of malignant colonic polyps: a population-based analysis of colonoscopic polypectomy versus surgery. Cancer 118(3):651–659. 10.1002/cncr.26340 21751204 10.1002/cncr.26340 PMC3193545

[10] Richards CH, Ventham NT, Mansouri D, Wilson M, Ramsay G, Mackay CD, Parnaby CN, Smith D, On J, Speake D, McFarlane G, Neo YN, Aitken E, Forrest C, Knight K, McKay A, Nair H, Mulholland C, Robertson JH, Carey FA, Steele R (2018) Scottish Surgical Research Group. An evidence-based treatment algorithm for colorectal polyp cancers: results from the Scottish Screen-detected Polyp Cancer Study (SSPoCS). Gut 67(2):299–306. 10.1136/gutjnl-2016-312201 27789658

[11] Leong K, Hartley J, Karandikar S (2017J) Association of Coloproctology of Great Britain & Ireland (ACPGBI): guidelines for the management of cancer of the colon, rectum and anus (2017) - follow up, lifestyle and survivorship. Colorectal Dis 19(Suppl 1):67–70 28632315 10.1111/codi.13706

[12] Johnstone MS, McSorley ST, McMahon AJ (2023O) Management of malignant T1 colorectal cancer polyps: results from a 10-year prospective observational study. Colorectal Dis 25(10):1960–1972 37612791 10.1111/codi.16716

[13] Grainville T, Bretagne JF, Piette C, Rousseau C, Bordet M, Cosson M, Lièvre A (2020A) Management of T1 colorectal cancers detected at screening colonoscopy: a study from the French national screening programme. Dig Liver Dis 52(8):909–917 32505572 10.1016/j.dld.2020.04.022

[14] Yeh JH, Tseng CH, Huang RY, Lin CW, Lee CT, Hsiao PJ, Wu TC, Kuo LT, Wang WL (2020N) Long-term outcomes of primary endoscopic resection vs surgery for T1 colorectal cancer: a systematic review and meta-analysis. Clin Gastroenterol Hepatol 18(12):2813–2823 32526343 10.1016/j.cgh.2020.05.060

[15] Williams JG, Pullan RD, Hill J, Horgan PG, Salmo E, Buchanan GN, Rasheed S, McGee SG, Haboubi N (2013) Association of Coloproctology of Great Britain and Ireland. Management of the malignant colorectal polyp: ACPGBI position statement. Colorectal Dis 2:1–38 10.1111/codi.12262 23848492

[16] Tanaka S, Saitoh Y, Matsuda T, Igarashi M, Matsumoto T, Iwao Y, Suzuki Y, Nozaki R, Sugai T, Oka S, Itabashi M, Sugihara KI, Tsuruta O, Hirata I, Nishida H, Miwa H, Enomoto N, Shimosegawa T, Koike K (2021A) Evidence-based clinical practice guidelines for management of colorectal polyps. J Gastroenterol 56(4):323–335 33710392 10.1007/s00535-021-01776-1 PMC8005396

[17] Symer M, Connolly J, Yeo H (2022) Management of the malignant colorectal polyp. Curr Probl Surg 59(5):101124. 10.1016/j.cpsurg.2022.101124. 10.1016/j.cpsurg.2022.101124 35568407

